# The Significance of Subclinical Epileptiform Activity in Alzheimer's Disease: A Review

**DOI:** 10.3389/fneur.2022.856500

**Published:** 2022-04-04

**Authors:** Emoke Anna Csernus, Tom Werber, Anita Kamondi, Andras Attila Horvath

**Affiliations:** ^1^School of PhD Studies, Semmelweis University, Budapest, Hungary; ^2^Neurocognitive Research Center, National Institute of Mental Health, Neurology and Neurosurgery, Budapest, Hungary; ^3^Faculty of Medicine, Semmelweis University, Budapest, Hungary; ^4^Department of Neurology, Semmelweis University, Budapest, Hungary; ^5^Department of Anatomy, Histology and Embryology, Semmelweis University, Budapest, Hungary

**Keywords:** Alzheimer's disease, subclinical epileptiform activity, epilepsy, neurophysiology, antiepileptic drugs

## Abstract

Hyperexcitability is a recently recognized contributor to the pathophysiology of Alzheimer's disease (AD). Subclinical epileptiform activity (SEA) is a neurophysiological sign of cortical hyperexcitability; however, the results of the studies in this field vary due to differences in the applied methodology. The aim of this review is to summarize the results of the related studies aiming to describe the characteristic features and significance of subclinical epileptiform discharges in the pathophysiologic process of AD from three different directions: (1) what SEA is; (2) why we should diagnose SEA, and (3) how we should diagnose SEA. We scrutinized both the completed and ongoing antiepileptic drug trials in AD where SEA served as a grouping variable or an outcome measure. SEA seems to appear predominantly in slow-wave sleep and in the left temporal region and to compromise cognitive functions. We clarify using supportive literature the high sensitivity of overnight electroencephalography (EEG) in the detection of epileptiform discharges. Finally, we present the most important research questions around SEA and provide an overview of the possible solutions.

## Introduction

Alzheimer's disease (AD) is the primary cause of cognitive deterioration with rapidly increasing prevalence among society (1). While currently curative therapy is not available, novel scientific studies highlighted modifiable risk factors and comorbid conditions serving as potential therapeutic targets (2, 3). Among these, the pathogenic role of epileptic activity has been proposed in animal studies showing that transgenic AD model animals present frequent epileptic seizures and aberrant neural hyperactivity (4, 5). These findings were corroborated by human experiments (6) highlighting that patients with AD or patients with mild cognitive impairment (MCI) have an elevated risk to develop epileptic seizures (7–9) leading to worse cognitive functions and increased progression of disease (10, 11), so their precise detection is a priority. However, the majority of seizures are nonmotor seizures with temporal lobe semiology; therefore, their detection could be difficult in patients with cognitive symptoms (7, 9). Recent reports also point to the possible importance of isolated epileptiform discharges, which occur without overt epileptic seizures in the pathogenesis of dementia (12, 13). In some neurophysiology studies, these discharges are named as subclinical epileptiform activity (SEA) (7, 12, 13). Epileptiform discharges without seizures are considered as benign variants in traditional epileptology that might lead to transient cognitive changes, but do not associate with persisting pathologic conditions (14). They frequently appear in healthy subjects and their incidence is increasing with age (15). While the negative effect of SEA on cognitive functions has been proposed in epilepsy studies since the 1990s (16–18), the phenomenon was not investigated in AD prior to the study of Vossel et al. (7). While a growing body of evidence suggests the common appearance of SEA in AD, there are numerous discrepancies and inconsistencies in reported results. The aim of this review is to summarize the current opinions on the role of SEA in AD and to propose further directions based on the edification of previous reports.

### Studies on SEA in AD

A search for “SEA” in PubMed on the 10th of December 2021 yielded 98 results between 2000 and 2021 and we selected papers for review based on the following inclusion criteria: (1) original peer-reviewed articles published in academic journals with impact factors; (2) full text is available in English; (3) prevalence or incidence studies in AD or MCI; (4) original research articles; (5) compared subject groups consist of healthy controls (HC) vs. patients with AD spectrum disease (AD or MCI); and (6) SEA was analyzed [studies analyzing epileptic discharges in AD patients with epileptic seizures (interictal discharges) were not selected]. Finally, eight studies (average impact factor = 6.46) matched the inclusion criteria (Table 1).

**Table 1:** Studies on subclinical epileptiform activity (SEA) in Alzheimer's disease (AD).

	**References**	**Condition**	**Number of subjects**	**Prevalence of SEA**	***P*-value**	**Frequency of SEA with EEG**	**Temporal/spatial distribution of SEA with EEG**	**Type of neuro-physiology study**
1.	Brunetti et al. (19)	AD/MCI/ HC	50/50/50	6.38% AD, 11.63% MCI 4.43% control	0.43	0.015- 0.025/ h	ND/ ND	Overnight video PSG+ MEG
2.	Vossel et al. (7)	AD+MCI	113	6%	ND	ND	ND/ ND	Daytime routine EEG
3.	Liedorp et al. (20)	AD/MCI/ other dementia	510/225/971	3% AD	0.07	ND	ND/ ND	30-min daytime EEG
4.	Vossel et al. (12)	AD/HC	33/19	42.4 vs. 10.5%	0.02	0.03–5.18/h	9.9% W, 25.7% N1, 64.4% N2-N3/ 43% left temporal, 29% left central, 14% right frontal, 14% bifronto-temporal	Overnight PSG+ MEG
5.	Horvath et al. (9)	AD	42	28%	ND	ND	ND/ ND	24-h ambulatory EEG
6.	Horvath et al. (13)	AD/HC	52/20	54 vs. 25%	0.01	0.29–6.68/h	8% W, 23% N1, 21% N2, 34% N3, 4% REM/ 52% left temporal, 22% right temporal, 26% bitemporal, 3% biparietal, 3% right frontal, 9% bifrontal	24-h EEG
7.	Lam et al. (21)	AD/HC	84	22 vs. 4.7%	0.02	1.5–3/ day	20% N1, 80% N2/ 85.7%, 28.6% bifrontal	24-h EEG
8.	Babiloni et al. (22)	AD+MCI/HC	32/32	41%	ND	ND	ND/ ND	resting state EEG

*The incidence of SEA varies among the studies between 3 and 54% in patients with AD probably due to the prominent differences in the methodology and reporting protocols. Sleep EEG was applied in five reports, while three reports used daytime EEG with a short-recording period. The spatial and temporal characteristic of SEA was analyzed only in three reports. Comparison with the incidence in healthy controls was reported only in 3 studies but the significantly elevated occurrence is constant among the findings. The exact incidence, characteristic, and significance cannot be properly estimated due to the large variety of the studies*.

*AD, Alzheimer's disease; MCI, mild cognitive impairment; HC, healthy control; SEA, subclinical epileptiform activity; ND, not defined; EEG, electroencephalography; MEG, magnetoencephalography; PSG, polysomnography; W, wakefulness; N1, 1st stage of sleep; N2, 2nd stage or sleep; N3, 3rd stage of sleep; REM, rapid eye movement sleep*.

According to these results, the prevalence of SEA varies from 3 to 54% among patients with AD and MCI. Most of these reports indicate a higher prevalence compared to HC' however, there are exceptions. The pivotal point to consider is the definition of SEA itself. The earliest studies published in 2013 did not clarify the neurophysiological definition of SEA (7). Liedorp et al. (20) referred the reader to a definition by the International Federation of Clinical Neurophysiologists recommendation from 1999 which did not define SEA separately (23). According to this guideline, epileptiform pattern or epileptiform discharge or epileptiform activity describes transients distinguishable from background activity, with a characteristic spiky morphology, typically, but neither exclusively nor invariably, found in interictal electroencephalographies (EEGs) of people with epilepsy. So, the guideline mentions that patterns might occur in people without epilepsy but does not define SEA as a specific category. In a decisive guideline article, Noachtar and Rémi defined SEA as paroxysmal EEG graphoelement (spikes or sharp waves), with 20–200 ms duration, with the disruption of background EEG activity followed by slow waves (24). Importantly, these graphoelements do not associate with the occurrence of epileptic seizures. The above criteria were applied in the study of Vossel et al. (12) and in the studies of Horvath et al. (9, 13). Some recent studies have defined SEA more strictly by focusing on the abrupt change in polarity. This meant to emphasize the multiphasic character of spikes and sharp waves, considering a sequence of a minor positive, major negative, and second minor positive component as a marker of epileptiform discharge (19, 21). In their most recent study of Lam et al. (21), a panel was created from nine clinical experts to evaluate morphology and existence of SEA in the study data and a consensus of > six experts was needed for the decision. They also conducted supplementary statistical analysis to avoid major flaws of interpreter variations (25). In the report of Brunetti et al. (19), dedicated software was used for spike detection after which discharges were manually revised. The most complex reporting protocol was applied in the study of Babiloni et al. (22) based on two current reference guidelines (26, 27). SEA needed to fulfill at least four of the following six criteria: (1) Di- or triphasic waves with sharp or spiky morphology; (2) different wave durations from the ongoing background activity, either shorter or longer; (3) asymmetry of the waveform: a sharply rising ascending phase and a more slowly decaying descending phase or vice versa; (4) transient component of SEA followed by an associated slow after wave; (5) background EEG activity surrounding SEA disrupted by the presence of the SEA; and (6) distribution of the negative and positive EEG potentials on the scalp suggesting a focal source of the signal in the cortex, corresponding to a radial, oblique, or tangential orientation of the (dipole) source. In summary, all the studies proposed that SEA seems like an epileptic transient, but the patients do not have epileptic seizures, so the importance of this activity is not clarified.

Interestingly, the clinical significance of SEA is barely estimated. The SEA positive and negative groups are compared with neuropsychology only in a few studies with different test batteries. No differences were reported with the Mini-Mental State Examination (MMSE) (9, 13, 21, 22) and with the Clinical Dementia Rating Scale (CDR) (21). However, patients with SEA had significantly lower scores on memory measured with the Addenbrooke Cognitive Examination (ACE) in the recent study of Horvath et al. (13). The same study highlighted that SEA was also associated with higher VLOM ratios (scores of verbal fluency + language subdomains/orientation + memory subdomains), which is a typical neuropsychological appearance of AD. Longitudinal measurements were applied only in two studies: Vossel et al. (12) executed a 1.3–1.7-year long follow-up and Horvath et al. (13) re-evaluated the subjects after a 3-year long period. Both the studies showed a significantly faster cognitive decline during the follow-up among AD patients with SEA. Unfortunately, no comparisons are available applying other cognitive biomarkers including cerebrospinal fluid (CSF) amyloid and tau levels or structural MRI and PET findings.

For a better understanding of SEA, we need to address the following question: what might be the origin of a wide range of prevalence? One possibility could be that different neurophysiological methods were used in the various studies. As research has shown, the routine 20–30 min EEG is inferior to the long-term 24-h EEG in capturing epileptiform discharges (7, 28, 29). A strong correlation (*r* = 0.972) was described between the length of the EEG and the detection possibility of epileptiform discharges (29) and it was also shown that SEA could be detected predominantly during sleep (9, 12, 29). In the experiment of Lam et al. (21), 97.6% of SEA was detected in nonrapid eye movement sleep (REM) sleep. In the study of Horvath et al. (13), the occurrence of SEA was mainly related to N2 (31%) and N3 (34%) sleep, while 23% of the spikes occurred in N1, 8% of the spikes occurred in awake state, and only 4% of the spikes occurred during REM sleep. Similar results were presented in the study of Vossel et al. (12); 9.9% of SEA was found in wakefulness, 25.7% of SEA was found in N1, and 64.4% of SEA was found in deeper (N2 and N3) sleep stages. The study of Brunetti et al. (19) did not confirm these findings, since in their report SEA frequency did not differ between the various sleep stages. Earlier studies mostly used 30-min long routine EEGs (7, 20) and these provided the lowest prevalence values. In most recent studies that utilized long-term EEG (9, 12, 13, 19, 21) either 24-h ambulatory EEG or polysomnography, constantly depicted higher prevalence of SEA. The length of EEG registration is decisive in the calculation of SEA frequency. Short-term EEGs cannot be used to calculate the density of epileptiform signals. In total, 24-h EEG studies reported the average frequency of SEA with a large variety ranging from 0.03 to 6.68/h, suggesting that large individual differences in the number of SEA may appear between patients with AD (Table 1). The exact proportion seems to be important considering that density might associate with the progression of cognitive decline (13).

Some studies included 1-h resting magnetoencephalography (MEG) results, such as Brunetti et al. (19) who found a higher detection rate with MEG (AD 33.3 and HC 10.5%) compared to long-term EEG (AD 6.4 and HC 4.4%). Similar results were highlighted in the observation of Vossel et al. (12) showing higher incidence with MEG than long-term EEG (42.4% with MEG vs. 33.3% with EEG) and larger density (1–20/h with MEG vs. 0.03–5.18/h with EEG). MEG observations also support the individual characteristic of SEA frequency among patients with AD. The higher sensitivity of MEG might also indicate that SEA is more frequently generated in deeper cortical areas, from where electrical signal detection is impeded by surface EEG electrodes. This idea is supported by two recent reports detecting epileptiform activity via foramen ovale electrodes in patients with dementia and epilepsy (30, 31). Of note, in the study of Lam et al. (30), 95% of epileptiform activity could not be detected on the scalp registration. A combination of EEG and MEG can aid the detection of abnormal electrical activity arising from the amygdala and hippocampus, which are known to play a crucial role in memory consolidation (32).

Detection methods seem to be important from the viewpoint of the spatial location of SEA as well. The dominance of left-sided appearance is consistent across the studies. In the study of Horvath et al. (13), 52% of SEA showed left temporal occurrence. In the two studies of Vossel et al. (7, 12), the left temporal presentation was more common than the right temporal presentation. Interestingly, MEG showed the opposite results in the same study showing right dominant appearance, but authors state that the reason is unclear (12). Spatial distribution was also analyzed in the study of Lam et al. (21) showing that SEA was present in 85.7% over the left temporal region. Interestingly, the same study also analyzed AD patients with epileptic seizures and highlighted those epileptic discharges (which do not correspond to SEA) in epileptic patients show different patterns where right temporal occurrence was predominant (42.9%). These findings draw attention to the importance of the spatial distribution of epileptiform activity and clarify the need for source localization with MEG or low-resolution brain electromagnetic tomography (LORETA). While these techniques were applied in three studies (12, 19, 22), their accuracy was not compared.

Other aspects that carried inconsistency in the detection of SEA were the various age, education level, and pharmacological status of patients included in the reviewed studies. As per patient age, in the recent study of Horvath et al. (13), the AD subgroup was considerably older than the control group (75 vs. 67 years). In the study of Lam et al. (21), controls were also significantly younger than AD patients with SEA (72 vs. 76 years). Similar but nonsignificant differences are presented in all the reviewed studies. Furthermore, two studies have suggested that a higher level of education might be a risk factor for SEA (7, 9), while others were not able to confirm these results. Since education is not highlighted as a risk factor for the development of epileptic discharges in the epilepsy literature, a possible explanation is the extensive recruitment of highly educated patients in sophisticated neurophysiological studies, as we can depict this in the study of Lam et al. (21). Patients' medication status such as taking psychoactive drugs is of crucial significance, yet a difficult one to optimize, as most of these patients are prone to rebound depression and adverse effects in case of withdrawing medication for the sole purpose of research. Yet antidepressants, acetylcholinesterase inhibitors, memantine, or atypical antipsychotics might decrease the seizure threshold and might distort the results (33). Noticeably, all the studies that were included in our analysis differ significantly in the medication regime. In the studies of Horvath et al. (9, 13), antidepressant use served as an exclusion criterion, 100% of patients were taking acetylcholine inhibitors and ~20% of patients were on memantine therapy. In the study of Vossel et al. (12), 50% of patients were on antidepressants and 21% of patients were on cholinesterase inhibitors. In the study of Lam et al. (21), 44% of the patients were taking cholinesterase inhibitors and memantine was prescribed for 20%. It is noteworthy that patients in the study of Brunetti et al. (19) were drug naive. Others did not clarify the therapeutic regimen so clearly.

A further concern is the occasional lack of control group in these studies. A HC population is included in four studies (12, 13, 19, 21). The observation of Brunetti et al. (19) investigated 50 probable AD, 50 MCI due to AD, and 50 healthy aging patients and have found no significant dissimilarity among the groups in terms of SEA (6.38% in AD, 11.63% in MCI, and 4.43% in HC). These results are conflicting with Vossel's study (12) (42.4% in AD and 10.65% in HC), Lam's study (21) (22% in AD and 4.7% in HC), and Horvath's study (13) (54% in AD and 25% in HC). Brunetti et al. note that their study populations were significantly older compared to the other three studies, which leads back to our consideration of patients' age discussed above. The large variety of SEA in HC is another pitfall. Data range between 0.1 and 26% (12, 13, 15, 19, 21, 34–36) and the large variety and the significant of SEA in healthy individuals still wait for the explanation. A very recent review proved to be helpful to clarify the concerns around the prevalence of SEA in AD (37). The authors presented a meta-analysis of SEA in patients with dementia where pooled estimate effects were calculated using random-effects models from five studies. They included 721 patients with AD and of these 44 had SEA. The pooled cumulative incidence rate and prevalence rate of SEA among patients with AD were 21.41 and 9.73%, respectively. The incidence rate was significantly higher than in controls (5.54%) The calculated relative risk of SEA in AD was 2.69. The major finding of the analysis is that SEA studies suffer from prominent inconsistencies concerning the neurophysiological methodology.

Due to the large variety in the methodology of studies on SEA in dementia, the significance of SEA is still questionable. However, interventional studies targeting epileptiform discharges might elucidate further considerations.

### Current Application of SEA in AD Drug Trials

Neuropharmacology has been an advancing field in the past decades, with large amounts of research effort being diverted toward the direction of antiepileptic drugs (AED) in the use of neurocognitive disorders. These studies have a large range of clinical focuses, many works on reducing epileptic activity; others prioritized improving the psychosis and agitation in patients with different stages of dementia, while some focused on delaying the cognitive decline. Results show a wide array of outcomes, ranging from showing no significant delay in cognitive decline over a 2-year period of trial medicine therapy (38), through demonstrating cognitive improvement with levetiracetam (LEV) in specific cognitive areas such as attention and verbal fluency (39), to even proving in a retrospective observational study that older adults taking AEDs showed a significantly higher relative risk of developing dementia than those not taking AEDs (40).

Searching published and ongoing clinical trials in the period 2000–2021 using “antiepileptic” and “dementia” as keywords on Clinicaltrials.gov yielded 31 results, while searching Pubmed for “antiepileptic,” “dementia,” “randomized,” and “therapy” offered 149 studies on the 10th of December 2021. In this section, we highlight studies only where epileptiform discharge was used as an eligibility criterion, diagnostic criterion, or outcome measure. One published study and three ongoing trials matched the above selection criteria.

In a randomized, double-blind, placebo-controlled, phase 2a crossover clinical trial (41), 125 mg oral LEV was administered twice daily for 4 weeks followed by 4 weeks of no drug use and another 4 weeks of placebo administration (group A). The other group was assigned to reverse order administration (group B). AD patients with SEA were compared to patients with AD without SEA. Both the groups consisted of 17 participants. SEA was detected with overnight EEG and 1-h MEG-EEG protocol. The exact definition of SEA is not clarified in this study, but we can assume that the same was applied as in the previous reports (7, 12) SEA was detected in 32.3% of patients and EEG showed superiority in detecting SEA compared to MEG. EEG recording revealed epileptiform discharges mostly in the left temporal region, while MEG showed random distribution. The frequency of SEA was similar (~2.5/h) to the previous study of Vossel et al. (12). Baseline characteristics of SEA negative and SEA positive patients did not show significant differences. Outcome measures were set to examine the changes in executive functions (*via* NIH EXAMINER computer battery), in epileptiform activity frequency, in cognitive functions [measured via virtual route learning task, Stroop interfere naming subscale, Alzheimer's Disease Assessment Score-Cognitive Subscale (ADAS-Cog)], in behavior and level of disability, and in MEG power spectrum measures (along with MEG functional connectivity measures). While no significant difference was found in most primary outcomes (including the reduction of SEA), LEV was found to be well tolerated and significant improvement in performances of Stroop naming task (+7.4 points in SEA+ vs. 0.3 points in SEA-group; *p* = 0.046) and spatial memory task (measured with virtual route learning; *p* = 0.02) was demonstrated in the group of AD patients with SEA. Noticeably, LEV also improved global cognitive performance in the SEA positive group measured with ADAS-Cog scores and NIH EXAMINER scores, but the results were not significant.

An on-going randomized, cross-over trial with 85 early AD participants conducted by Mouhsin Shafi et al. (identifier: NCT03875638; estimated to finish in August 2023), set out to explore the relationship between abnormalities of brain network function, cognitive dysfunction, cortical hyperexcitability, and alterations of all these with administrations of low dose (125 mg twice daily) and high-dose (500 mg twice daily) LEV. Cognitive outcomes are measured by a neuropsychological test battery looking at mean z-score changes relative to baseline. Electrophysiological outcome measures of cortical excitability will be examined via transcranial magnetic stimulation (TMS) resting motor threshold, while network excitability measures will utilize TMS-evoked EEG hypersynchrony with stimulation of the parietal cortex. The presence or absence of epileptiform discharges is defined as the baseline measurement to determine cortical hyperexcitability. SEA is measured with high-density EEG and 24-h ambulatory EEG. Patients with epilepsy or with a history of epileptic seizures are excluded from the study.

An interventional single group study utilizing 65 patients between the ages of 60–90 was designed to determine if LEV changes the severity of neuro-psychiatric symptoms in patients with AD exhibiting SEA compared to patients with AD without epileptiform discharges (identifier: NCT04004702; estimated date to finish is Dec 2024; PI: Timothy R Malone). AD patients with epileptic discharges receive on a twice-daily regimen 500 mg LEV. SEA is captured with EEG. The definition of SEA or the exact neurophysiological methodology is not clarified in the available study protocol. The study plan involves following the participants for 1 year and the comparison of the results to the baseline of the next outcome measures: neuropsychiatric inventory score, CDR-sum of boxes (CDR-SOB), clinical AD severity as Alzheimer' Disease Cooperative Study Clinical Global Impression of Change (ADCS-CGIC), MMSE, and EuroQol 5-dimension (EQ-5D). The reduction of SEA does not serve as an outcome measure. Positive results would point to a possibility to utilize anti-epileptic treatment to slow down the neural decline.

Another study is set to assess the utility in treating AD with LEV (identifier: NCT02002819, PI: Keith A Vossel, estimated to finish in December 2021). This cross-over randomized trial sets its outcome measures as the change in SEA frequency (investigated using M/EEG), the change in cognitive function with Stroop test and Alzheimer Disease Assessment Scale (ADAS-cog/ADAS-ADL/ADCS-CGIC), changes in behavior and level of disability (CDR), neuropsychiatric inventory (NPI), changes in cognitive function as measured by virtual-navigation task, MEG Power spectrum measures and MEG functional connectivity measures, together with the blood serum of LEV and prolactin. 36 subjects aged 45–80 will be followed for 12 weeks. In the initial patient examination, any epileptic activity will be determined and used as a baseline for comparison to assess treatment effects. The drug regimen consists of 4 weeks LEV (125 mg) therapy followed by 4 weeks of no drug use that is followed by 4 weeks of placebo administration, with a second group using the reverse order of this regimen. Although the trial is not yet over (estimated termination was set as December 2021), the latest update (September 2020) showed that while no significant change was found in the primary outcome measures, LEV did improve performance on executive function tasks and spatial memory in patients who exhibit both AD and epileptiform activity, showing a promising route of addressing the cognitive decline in AD. Some results but probably not the entire dataset have been already published in 2021 (41).

We can conclude that while a growing number of studies analyze the utility of AED in dementia, only a few of them apply neurophysiologic methods and exploit the benefit of epileptiform activity as a biomarker for the selection of a proper target population. LEV is overrepresented in these trials and while there are many other AED in clinical trials of AD, none of them applies SEA as a biomarker. However, the reviewed studies consistently suggest the beneficial impact of this methodology in drug trials.

### Discussion and Future Perspectives

Following the development of the last two decades investigating seizure activity in dementia and MCI (10), the focus of research slowly has started steering toward patients with AD without an epileptic history. Although the number of studies focusing on SEA is increasing, the comparison remains challenging due to not standardized methodology. Since an explicit tendency is visible suggesting its importance in the pathomechanism of AD, there is a clear need for further studies to answer three important questions: (1) What is neural mechanism behind SEA?; (2) what is the importance of SEA?; (3) How should we diagnose SEA (diagnostic criteria and applied neurophysiological method)?

Novel observations started to draw more attention to SEA since increased incidence is detected consistently in diseases affecting cognitive functions, including AD (42). Based on recent reviews, the leading research sites agree that SEA is a characteristic EEG hallmark of AD-related cortical hyperexcitability (10, 42–45). It seems also feasible that the imbalance between excitatory and inhibitory regulation propagates from the dentate gyrus to the hippocampus and later to cortical structures explaining the dominant occurrence of SEA in the temporal regions (43). Increase in excitability associates with the hypersynchronous states of neural networks. The strongest synchrony among neuron populations is achieved during slow-wave sleep (46), possibly explaining the increased occurrence of SEA in deep sleep. Its pathologic role in AD is postulated by numerous reports suggesting that hyperexcitability might lead to increased neurodegeneration due to excitotoxicity, it changes the long-distance neural network connections and disrupts sleep-related memory consolidation (42). These concepts are supported by two of the reviewed studies demonstrating that appearance of SEA is not benign since it associates with increased progression of cognitive decline (12, 13) and with lower cognitive functioning (13) and there is a clear correlation between the higher number of discharges and faster disease progression (13). These observations are reinforced by a current report showing that SEA in AD is characterized with the prominent reduction in the alpha band and increase in delta-theta band coherence corresponding to an accelerated longitudinal decline in MMSE scores (47). Considering the above-described details, the answer for the first question is that SEA might be the neurophysiological representation of increased excitability and might contribute to the pathologic process of neural loss in AD.

The importance of SEA seems to stand on two pillars: its potential application as a marker of (1) epileptogenesis and (2) cognitive deterioration. Fortunately, seizures in AD are frequently indicated by EEG abnormalities (interictal epileptic discharges) (7, 9), but they show a similar appearance as SEA. Thus, the distinction of SEA and interictal activity is challenging. The decision might be driven by heteroanamnestic data obtained from caregivers focusing on possible seizure-like episodes and by the precise acquisition of medical history regarding the conditions lowering seizure threshold (history of central nervous system infection, alcohol and drug dependency, head trauma with loss of consciousness, antipsychotic drug use, renal and kidney failure, etc.). Another possible method is the precise analysis of the neurophysiological characteristics of the epileptiform discharges. The analysis of Lam et al. (21) highlighted important differences between SEA and epileptiform discharges associated with seizures: (1) 97.6% of SEA occurred in non-REM sleep while 28.7% of interictal discharges were present in REM and wakefulness; (2) 85.7% of SEA was lateralized to the left temporal area while 42.9% of interictal discharges showed right temporal appearance; (3) the infrequent occurrence of spikes (~ <10/24-h) was characteristic for SEA while the ~ 1/h occurrence for typical for interictal activity. While further observations are needed to clarify these findings, the same spatial and temporal distributions are reported in the other reviewed studies. Another important supportive reason for the precise detection of SEA is the proper selection of patients benefiting from the use of AED. While there are numerous AED studies in AD, they show ambiguous results querying the importance of an antiepileptic regime in dementia. Precise selection of patients with obvious signs of cortical hyperexcitability as SEA might provide an ideal target population. This is reinforced by the study of Vossel et al. (41) observing that only patients with SEA presented better cognitive scores following the administration of LEV. Based on these findings, the clear exclusion of epilepsy and the possibility for patient-driven precision drug therapy are the advantages of the detection of SEA.

How should we diagnose SEA? First, we need to decide what neurophysiological tools to use considering the cost-benefit ratio. Routine EEG is the most readily available and cheapest possibility, while MEG is the most expensive technique. The selected method needs to capture the spatial and temporal features of SEA since these features are important in the detection of epilepsy (21) and show strong associations with the disease course (13). The frequency of SEA seems to be a crucial parameter as well. Routine, 30-min daytime EEG with 21 electrodes does not fulfill these aims since most of the discharges occur in sleep (12, 13, 21). High-density EEG (64 or 128 electrodes) has a good spatial resolution and it is ideal for source localization, but the appropriate placement of the electrodes is time-consuming and it is more expensive than routine EEG. MEG has a great spatial and temporal resolution, but it is barely available, extremely expensive, and needs specially trained staff. Overnight EEG with 21 electrodes seems to be superior in the diagnosis of SEA since it is a well-tolerated and cheap alternative, able to capture SEA in sleep, and helps the localization (12, 19, 21). Associated application of LORETA provides an opportunity for source localization as well (22). Furthermore, ambulatory solution is also possible without the need for hospitalization (9, 13). When it comes to the diagnosis, the definition of SEA needs to be considered as well. The reviewed studies applied different guidelines (23–27) and resulted in various incidence values, which results might have been influenced by the above discrepancy. In our opinion, the best neurophysiological characterization of SEA cannot be provided in the current phase of research. Altogether, we can propose a clear need for unified EEG protocols for the definition of SEA. Panel discussions by leading experts, symposia to provide the definition, establishment of a common library of SEA graphoelements, and publishing a consensus paper would be the ideal direction to facilitate automated detection methods and further research on AD-related epileptiform activity.

**Figure 1:**
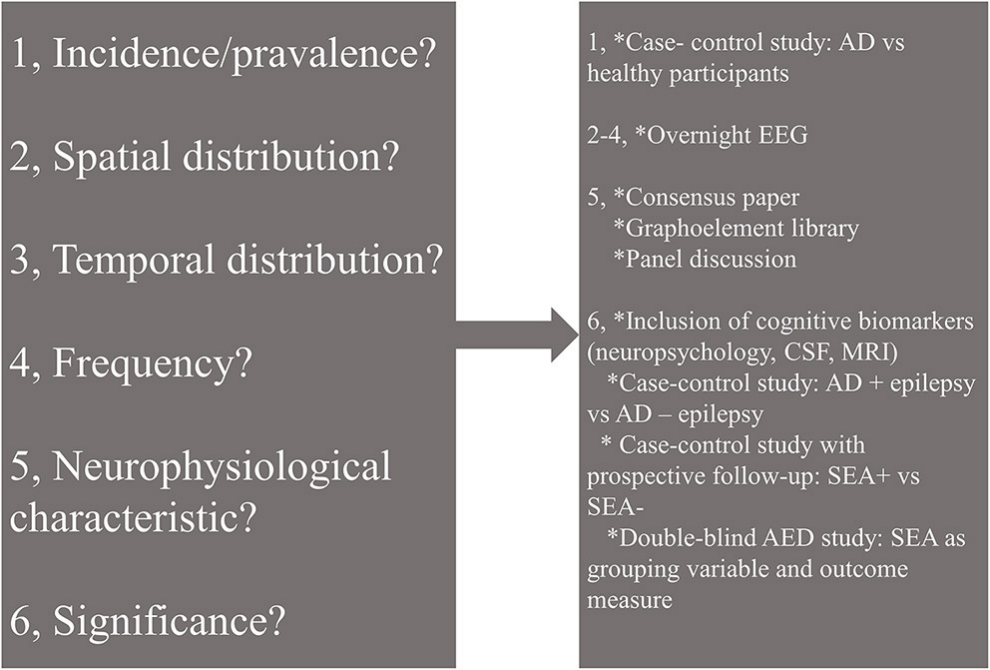
Open questions (left) and possible solutions (right) in the research of subclinical epileptiform activity (SEA) in Alzheimer's disease. AD, Alzheimer's disease; EEG, electroencephalography; CSF, cerebrospinal fluid; MRI, magnetic resonance imaging; AED, antiepileptic drug; SEA, subclinical epileptiform activity.

In conclusion, hyperexcitability represented by SEA is a relatively newly recognized contributor to neurodegenerative processes. Reports on SEA in AD showed conflicting results regarding the incidence probably due to the various methodologies. Some of the characteristic features of SEA seem to be unified among the reviewed studies: (1) left temporal appearance; (2) dominant occurrence in slow-wave sleep; (3) association with worse cognitive performance. These features seem to be useful in the differentiation of SEA and AD-related epilepsy. While a growing body of evidence highlights the possibility of AED use in dementia, SEA is barely applied as an outcome or inclusion criterion in drug trials. However, based on some reports, appropriate inclusion criteria help the precise detection of target groups. Further studies on SEA might illuminate details of the pathophysiology of AD and accelerate drug discoveries (Figure 1). While overnight EEG seems to be the ideal tool for the detection, there is a clear need for unified neurophysiology guidelines or consensus papers to stimulate research on AD-related epileptiform discharges.

## Author Contributions

EC and TW performed the literature search, data acquisition, and wrote the summary of the analyzed studies. AH and AK were responsible for the concept of the manuscript, for the discussion, and conclusion of the major findings of the reviewed reports. All authors contributed to the article and approved the submitted version.

## Funding

This study was supported by the National Brain Research Program I, II (KTIA_NAP_13-1-2013-0001; 2017-1.2.1-NKP-2017-00002), the Hungarian Scientific Research Fund 2019 of the National Research, Development and Innovation Office (PD- 132652), and the Janos Bolyai Research Scholarship of the Hungarian Academy of Sciences (bo_78_20_2020). This is an EU Joint Programme- Neurodegenerative Disease Research (JPND) project. The study is supported through the following funding organization under the aegis of JPND- www.jpnd.eu (*National Research, Development and Innovation, Hungary*, 2019-2.1.7-ERA-NET-2020-00006).

## Conflict of Interest

The authors declare that the research was conducted in the absence of any commercial or financial relationships that could be construed as a potential conflict of interest.

## Publisher's Note

All claims expressed in this article are solely those of the authors and do not necessarily represent those of their affiliated organizations, or those of the publisher, the editors and the reviewers. Any product that may be evaluated in this article, or claim that may be made by its manufacturer, is not guaranteed or endorsed by the publisher.
